# Supplementary material for: Probiotic Monotherapy with Lactobacillus reuteri (Prodentis) as a Coadjutant to Reduce Subgingival Dysbiosis in a Patient with Periodontitis
Source: Int J Environ Res Public Health. 2022 Jun 26;19(13):7835. doi: 10.3390/ijerph19137835 (PMC9265494; doi:10.3390/ijerph19137835)
Supplement: Supplementary file 1 [file ijerph-19-07835-s001.zip › ijerph-1733684-supplementary.pdf]

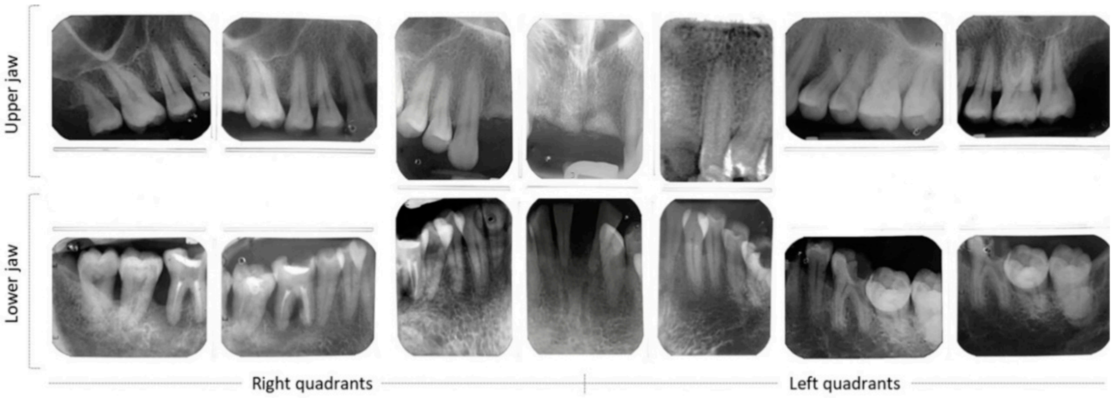

Figure S1. Periapical radiographs of the full mouth. The patient had 25 teeth, with a periodontal diagnosis of generalized periodontitis, stage IV, grade B [4]. The missing teeth observed at baseline (T0) correspond to the upper central and lateral incisors, the lower left incisor, and the upper first molars.

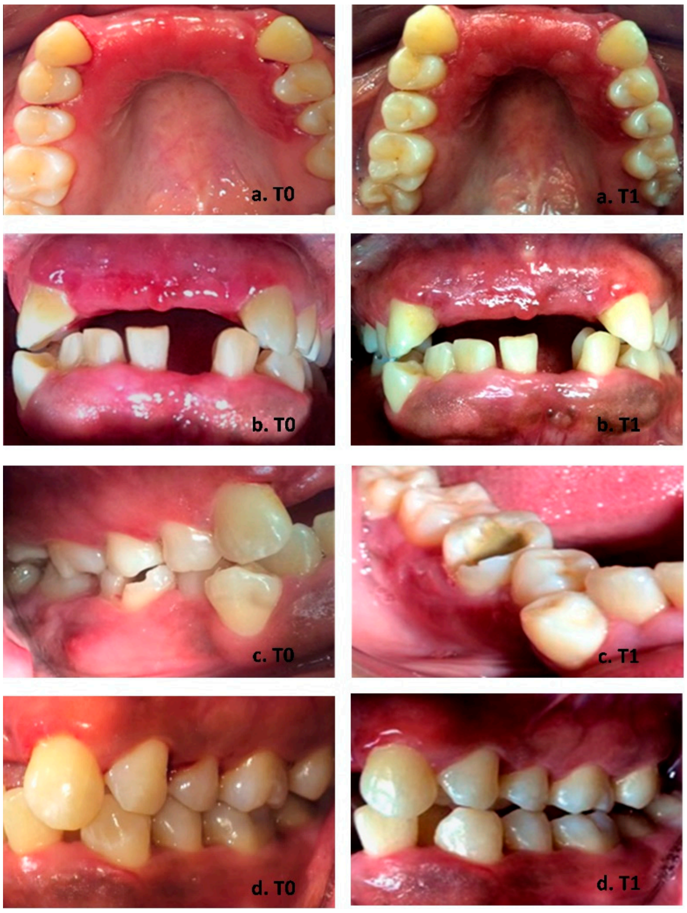

Figure S2. Representative clinical images. T0: baseline and T1: one month of L. reuteri Prodentis consumption. a. Occlusal view; b. Upper incisors region; c. Tooth 46; and d. Left molars region.
